# The perceived determinants and recommendations by mothers and healthcare professionals on the loss-to-follow-up in Option B+ program and child mortality in the Amhara region, Ethiopia

**DOI:** 10.1186/s12879-020-05583-6

**Published:** 2020-11-23

**Authors:** Mesfin Wudu Kassaw, Samuel T. Matula, Ayele Mamo Abebe, Ayelign Mengesha Kassie, Biruk Beletew Abate

**Affiliations:** 1 Department of Nursing, Woldia University, College of Health Science, P.O Box 400, Woldia, Ethiopia; 2 Faculty of Health Sciences, School of Nursing, University of Botswana, Gaborone, Botswana; 3 Department of Nursing, Debre Berhan University, College of Health Science, Debre Berhan, Ethiopia

**Keywords:** PMTCT, Exposed infants, Amhara, LTFU, Mortality

## Abstract

**Background:**

The third United Nations Sustainable Development Goal includes a commitment to end AIDS-related death by 2030. In line with the Goal, Option B+ programs hold a great promise for eliminating vertical transmission of HIV. Option B+ was introduced in 2013 in Ethiopia. The Global Plan identified Ethiopia as one of 22 high priority countries requiring improvement in prevention of mother to child HIV transmission services. Despite HIV treatment being free in Ethiopia, only 59% of children are on treatment. The discrepancies in high uptake of Option B+ and low numbers of children in Ethiopia can be attributed to Loss-to-follow-up, which is estimated from 16 to 80%. While LFTU is expected in the region, no-to-minimal evidence exists on the magnitude and its determinants, which hampers the development of interventions and strategies to reduce LFTU. The purpose of this study is to explore perception of mothers and healthcare providers on determinants of and recommendations to reduce LTFU and HIV exposed infants’ mortality.

**Method:**

Explorative, descriptive qualitative study conducted in five zones of Amhara region. The sample consisted of mothers enrolled to the option B+ programs at the five referral hospitals PMTCT departments, nurses and midwives working in those departments, and HIV officers in zonal departments. Data were collected in 2019 using in-depth interviews. Data were analyzed using content analysis and deduced to themes.

**Results:**

Overall, nine themes were identified from the interviews. Five themes represented the determinants of LTFU and mortality while four themes addressed the recommendations to reduce LFTU among mothers and their infant mortality. The determinants themes centered on apathy, stigma and discrimination, poor access to services, healthcare providers behavior and attitudes, and social determinants of health. While recommendations themes suggested that improving access, capitalizing on psychosocial support, education and awareness, and empowerment.

**Conclusions:**

Social and structural issues are major contributors to low retention of mothers and death of children due to HIV. A multi-stakeholder approach, including structural changes, are required to support women and their children to ensure that individuals, communities and country enjoy the full benefits of option B+ and lead to an HIV free generation.

**Supplementary Information:**

The online version contains supplementary material available at 10.1186/s12879-020-05583-6.

## Background

After the United Nations Millennium Declaration, great gains were made in addressing the global AIDS pandemic [1]. The coordinated scale-up of antiretroviral medications worldwide has contributed to the increase in life expectancy due to the decline in HIV/AIDS-related deaths [2, 3]. The United Nations envisions that efforts towards the elimination of HIV and HIV-AIDS related deaths will continue through the Sustainable Development Goal (SDG) [4]. The main challenge to eliminate HIV in children is the high numbers of new infections. The main challenge to eliminate HIV in children is the high numbers of new infections [5]. Nearly 2.8 million children aged 0-19 years old were living with HIV at the end of 2019, and 880 children were newly infected by HIV, while 310 children succumbed to AIDS-related illnesses daily [6]. In the absence of antiretroviral therapy, the rate of HIV vertical transmission varied between 15 and 45% [5]. Despite high new HIV infections in children, the vertical transmission rate has decreased from 18% in 2010 to about 9% in 2018 in East and Southern Africa, with as low as 2% in some countries [7].

According to the UNAIDS 2019 data, Ethiopia has 690,000 people living with HIV, 23000 new HIV infections and 11,000 AIDS-related deaths in 2018 [7, 8]. Women aged 15 years and above constituted 410, 000 of people living with HIV, 12000 new infections and 5500 AIDS-related deaths, while children 0–14 accounted for 36,000 of people living with HIV, 2700 new infections and 1800 AIDS-related deaths in Ethiopia by the end of 2018 [7, 8]. Sixty-five percent of people living with HIV are on ART in Ethiopia, of which 65% are women aged 15 and above and 59% children 0–14 years [7, 8]. The magnitude of HIV among pregnant women attending antenatal clinic (ANC) in Ethiopia varies from 0.2% [9] to 12.1% [10]. Ninety-two percent of pregnant women living with HIV have access to ART, with an early infant diagnosis of only 60% [7, 8]. Overall, the new HIV infection rate is going down, and fewer people are dying from AIDS-related deaths. However, children aged 0–14 new infections are still considerably high, given the percentage of pregnant women on ART.

The Global Plan identified Ethiopia as one of the 22 high priority countries that need improvement of the Prevention-of-Mother-to-Child HIV transmission (PMTCT) services, due to the discrepancies between the percentage of women on ART and high rate of HIV infection among children 0–14 which is mainly driven by mother-to-child HIV transmission [11]. Option B+ is the primary vehicle for both providing ART to pregnant women and prevention of HIV transmission in children 0–14 in Ethiopia since 2013. According to the operational plan, under Option B+, all HIV-infected pregnant mothers will receive triple antiretroviral therapy (ART) drugs and will continue the treatment for the rest of their lives which reduces the risk for HIV transmission to children to about 2% or less as demonstrated in some countries and AIDS-related deaths for both mothers and children [9, 12, 13]. Additional benefits of Option B+ PMTCT includes simplification of ART to reduce errors and improve adherence, protection against MTCT in future pregnancies and avoiding stopping and starting of antiretroviral drugs [14]. With 92% of pregnant women on ART in Ethiopia, these benefits should be readily visible. Still, they are yet to be achieved mainly in some regions such as the Amhara Region in the northwest of Ethiopia.

According to Ethiopia Demographic & Health Survey (EDHS) results, the Amhara region hosts some hotspots for HIV infection [15]. The prevalence of mother-to-child HIV transmission in Ethiopia is estimated to be 11.4% while in Amhara region is estimated to be 9.56% (3.8 -12.4%) [16]. The prevalence of mother-to-child transmission is high compared to other regions in the country and countries with higher HIV risk that implemented the Option B+ program almost at the same time with Ethiopia were vertical transmission has been reduced to less than 1% [9, 13]. Therefore, Ethiopia, particularly regions such as Amhara region has not yet realized the full potential of Option B+ program. Therefore, challenges related with retention of pregnant women throughout the cascade of Option B+ in Amhara region and Ethiopia, in general, need to be investigated.

Loss-to-follow-up (LTFU) has been identified as a major impediment to the successful implementation of option B+ program in sub-Saharan Africa, leading to increased mother-to-child HIV infection [17]. The rate of loss-to-follow-up in most of the sub-Saharan countries range from as low as 9.6% to a high of 61% depending on the level of PMTCT cascade and settings [17]. The prevalence rate of LTFU is estimated to be between 9 and 14.8 per 1000 person-months of the option B+ cascade [18]. LTFU disrupt the Option B+ cascade and increase the risk of mother-to-child HIV transmission, and lead to poor health outcomes for both the mother and child including advancing of HIV stages and maternal and child mortality [18–20]. Evidence shows that younger age, lack of education, stigma and discrimination, failure to disclose HIV-status, long distances to reach health facilities, long waiting times, poor adherence to treatment, high CD4 count and WHO clinical stage I, II were associated with LTFU [21–23]. These factors vary by country, and sometimes the national factors are not similar to the district level factors [21–23]. Therefore, to successfully develop interventions that reduce LTFU, researchers need to focus on smaller scales such as district-level factors. Although the Option B+ program is critical in preventing both HIV infection and mortality of children and their mothers after enrollment to the program, data are scarce on the determinants of LTFU and eventually mortality of children in Ethiopia. Therefore, the objective of this study is to explore the perception of mothers and healthcare providers on determinants of and recommendations to reduce LTFU in the Option B+ program, and HIV exposed infants’ mortality in the Amhara regional state, Ethiopia.

## Methods

### Design, study setting and period

This study used an explorative, descriptive qualitative design [24, 25]. The study was conducted in five zones of the Amhara regional state, Ethiopia. Amhara region is located to the southwest of Ethiopia and has 15 administrative zones. The region has five referral hospitals that provide PMTCT services per the option B+ national guideline [26]. The referral hospitals were found in north Shewa zone (Debre-Birhan referral hospital, Debre-Birhan, Ethiopia), south Wollo zone, (Dessie referral hospital, Dessie, Ethiopia), North Gonder zone, (Gonder referral hospital, Gonder, Ethiopia), west Gojjam zone, (Felege-Hiwote referral hospital, Bahir-Dar, Ethiopia), and east Gojjam zone (Debre-Markos referral hospital, Debre-Markos, Ethiopia). The period for data collection was from March 21 to May 18, 2019.

### Population

The sample consisted of mothers enrolled in the option B+ programs at the five referral hospitals PMTCT departments in the region, nurses and midwives working in those departments, and HIV officers in the zonal departments. HIV-officers are healthcare professionals assigned to the zonal department to control and facilitate HIV prevention programmes.

### Inclusion and exclusion criteria

#### Inclusion criteria

The inclusion criteria for mothers were 1) the mother should be expecting a child, 2) HIV positive and enrolled in the Option B+ program at one of the 5-referral hospitals of the Amhara region between January 01/2018 and September 2019 3) Able to understand consent in Amharic. Inclusion criteria for health professionals were 1) working for at least 2 years as an HIV officer or 2) having 2 years or more working in any of the five-referral hospitals PMTCT departments.

#### Exclusion criteria

Mothers were not considered if 1) they had several co-morbidities, 2) having antiretroviral failure, while healthcare providers were not considered if working on a half-day basis.

#### Sampling and sample size

Purposive sampling was used for the selection of subjects. Purposive sampling was used to ensure that the sample was heterogeneous and richness in data to ensure rigor. A sample size of 46 subjects was selected. A total of 24 mothers enrolled in the PMTCT department, 12 nurses and midwives working in the PMTCT departments, and ten zonal HIV officers. The sample size was based on data saturation.

#### Ethics

An ethical clearance (permit number: WDU/IRB-0076/2019) was obtained from the Woldia University institutional review board committee. An official permission letter was obtained from zonal health departments and town administrators. Written consent was obtained from all participants, and when necessary, the interviews were terminated at the behest of participants. Permission for audio-taping was also sought before the interviews, and the tapes were kept securely. All transcribed data was de-identified.

#### Data collection tools and procedures

Data was collected through individual interviews with a member of the research team. The interview was audio-taped and lasted from one and a half to two and a half hours using open-ended interview questions. The interview checklist developed in English guided by qualitative interview guideline [27] (Additional file 1), and translated to Amharic by an independent person and translated back to English by an independent person to ensure consistency. The interview guide was pretested on seven participants in Debre-Tabore town and contributed to some adjustments to the interview guide. Interviews were conducted during the mothers stay at the hospital on a single occasion and during working hours in the health facility for health workers using the Amharic language. Trained data collectors collected data with at least one previous experience of qualitative data collection. Trained data collectors collected data with at least one previous experience of qualitative data collection.

#### Data processing, quality assurance and data management

Transcribers were trained from the data collectors. The interviews were transcribed by experienced and certified qualitative data transcribers and translators. Two independent transcribers listened to audio record and transcribed the interviews verbatim. The difference between the audio record and transcribed text was verified through the member check. The transcripts were then translated to English and verified through member check if it has captured the essence of their experience. The transcripts were then read by the researchers to obtain a preliminary understanding of the participants’ experiences and the context [28]. Where it was not clear, the researchers listened to the tapes and clarified with the transcribers. Data was organized and managed through the N-Vivo 10 software [29].

#### Data analysis

Thematic analysis was used for data analysis [30]. Two authors independently read and coded the transcripts. In the initial exploration of the interview; the whole interview was read to capture the essence of the entire transcription. The authors then read the first four interviews (2 from mothers, one from Nurses and Midwives, and one from HIV officers) and generated the initial codes which were compared, disagreements on the interpretation of meaning units, code labels and categories were discussed until agreement was reached. The final codes were confirmed with an additional third author.

Consequently, N-Vivo 10 software [31] was used to extract the data from the interviews, including expanding the codes when necessary, using sentences and text passages [27]. Following the initial coding, a manual inductive analytic approach was used to expand the extracted codes using open codes and notes [29, 30]. In the initial coding cycle [32], the meaning units emerged freely from the text and had been given a descriptive code label (Table 1). In the second coding cycle [27], codes were sorted into mutually exclusive categories and sub-categories (Table 2). The categories and sub-categories were revised several times to ensure that the contributors to mortality and LTFU reflected the participants’ perceptions [27]. Data saturation was reached when the redundancy in the responses was recorded at least ten times, and when no new concepts emerged regarding the perception of LTFU and mortality.

**Table 1:** Examples of meaning units, condensed meaning units and codes

In-text statements	Condensed meaning unit	Code
“..Most professional are not interested to work in the PMTCT department...”	Lack of commitment	Health facility related factors
“..Mothers might not visit the PMTCT, and failed to take medication to their home because of their family’s reaction...”	Fear of family response	Stigma and discrimination
“…I often do not have sufficient money for transportation and self-care, and I will miss appointments…”	Poverty	Socioeconomic status
“…In order to prevent LTFU, mothers should join local mother to mother groups…”	Admitted to civic societies	Psychosocial support, empowerment

**Table 2:** An example of category, sub-category and descriptive codes

Codes	Sub-category	Category
Lack of professionals in number Lack of professionals with better education Lack of counseling training for professionals	Shortage of adequately trained staff	Health facility related factors
Poor professional ethics in the PMTCT	Poor customer service	Health care providers attitudes
Mother should appreciate the signs that caused them to visit health facilities Mothers should be counseled to visit health facility together with their children for screening	Health seeking behaviors	Parental awareness

#### Trustworthiness

Cognitive interviews were used to ensure that the interview guide was reliable and valid in Debre-Tabore town, which is 50 km from Bahirdar, the capital city of the Amhara region. Checking was used at the transcription stage to ensure that the transcribers captured the participants viewed correctly and were there a difference. Member check was used to ensure that the transcripts were capturing the essence of their experiences. The credibility, dependability, conformability, transferability, and authenticity were used to ensure trustworthiness [29]. To ensure credibility [24], we selected a maximum variation sample to capture the range and variations of the first-hand perception of mothers and stakeholders [33–35]. The interviews had open-ended questions, allowing the participants to speak freely, using their logic [33]. Dependability [24] was guaranteed by recruiting participants who had experience or understanding the phenomenon at first hand and having a team of coders rather than individuals and use of field notes [25]. Conformability [24] was guaranteed by two authors independently coding the transcripts. Transferability [24] was sought by providing detailed descriptions of all aspects of the study, helping readers to judge whether the findings would be applicable in other contexts. To ensure authenticity [36], the findings reflect multiple realities and differences in functional ability at each phase of the trajectory.

## Results

### Demographic characteristics of study participants

Forty-six participants completed interviews between March 10 and April27/ 2019 against the 57 expected interviews due to data saturation. The sample consisted of mainly females (73.9%) with age ranging from 20 to 59 years and 30–39 age groups being the modal age group (31.1%). Mothers and health professionals constituted 73.91 and 26.09% respectively. More demographic characteristics are presented in (Tables 3 and 4).

**Table 3:** The demographic characteristics of mothers in northwest Ethiopia

Variables	Categories	Frequency (***n*** = 24)	Percent	Mean
Mean age				36.21
Age group	20–29	7	29.17	
	30–39	13	54.17	
	40–49	4	16.66	
Mothers’ parity	1st Pregnancy	4	16.67	
	2nd Pregnancy	12	50	
	More than 3 pregnancies	8	33.33	
Mothers HIV+ Status	New (during antenatal care)	5	20.83	
	Known on ART	19	79.17	
	Known not on ART	0	0.00	
Mothers educational status	Primary	9	37.5	
	Secondary	5	20.83	
	College/university	10	41.67	
Profession	Housewife	11	45.83	
	Government employee	6	25	
	Merchant	7	29.17	
Place of participants	Debre-Markose	4	16.67	
	Gondar	6	25	
	Bahir-Dar	5	20.83	
	Dessie	4	16.67	
	Debre-Berhan	5	20.83

**Table 4:** The demographic characteristics of health care providers in northwest Ethiopia

Variables	Categories	Frequency (***n*** = 22)	Percent	Mean
Gender	Male	9	40.9	
	Female	13	59.09	
Type of health facility that of participants work	HIV officer at zonal health department	10	45.45	
	Professionals at the included hospitals	12	54.55	
Mean age	Hospital Health professionals HIV Officer at zonal health department			32.17 43
Age group	20–29	4	18.18	
	30–39	9	40.91	
	40–49	6	27.27	
	50–59	3	13.64	
Profession	Nurse	7	31.82	
	Midwife	11	50	
	Physician	0	0.00	
	Public health expert	4	18.18	
Work experience	2–4 years	12	54.55	
	5–8 years	6	27.27	
	More than 8 years	4	18.18	
Education status	Certificate	0	0.00	
	Diploma	6	27.27	
	Degree	16	72.73	
Place of participants	Debre-Markose	4	18.18	
	Gondar	5	27.73	
	Bahir-Dar	5	27.73	
	Dessie	4	18.18	
	Debre-Berhan	4	18.18	

### Themes

The results were categorized into two major groups; first addressing determinants of LFTU and child mortality, and secondly, recommendations to reduce LFTU and child mortality due to HIV in the Amhara region. Five (5) themes were identified as determinants of LTFU and child mortality, while four (4) themes were deduced as recommendations to minimize LTFU and child mortality (Table 5).

**Table 5:** Categories and Themes

Group	Determinants of LFTU and Child Mortality	Recommendations to prevent LFTU and child Mortality
**Themes**	Apathy	Access
	Stigma and discrimination	Psychosocial support
	Access	Education & awareness
	Health care providers behavior & attitudes	Empowerment
	Social determinants of Health	

### Determinants of loss to follow-up and child mortality

Determinants of loss to follow-up and child mortality are categorized into five themes; 1) Apathy- addressing parents’ attitudes towards their child’s health and willingness to commit to option B+ program, 2) Stigma and discrimination- capturing the parents’ fear of isolation from family and the community, self-blame and lack of disclosure of the status. 3) Access - elucidating on physical barriers to access such as distance to ART centers and resource shortages in ART centers. 4) Health care providers behavior and attitudes- highlight the lack of commitment and negative attitudes exhibited to PMTCT users by healthcare providers in the region, and 5) social determinants of health- discussing how poverty in the region lead to loss to follow-up and child mortality.

**Apathy** “*… Sometimes I missed the time of my follow up without any apparent reason, but when health care professionals contacted me through the phone, I came back again …*” M2.

Both parents and health care providers indicated that mothers’ top following up with option B+ without any valid reasons despite being aware of the benefits it carries for the child. Lack of concern was also identified as a major factor in poor child nutrition and consequently leading to illnesses and death. Mothers did not care for their children health despite being aware of the probability of the risk of being exposed to HIV. Mothers also opted for mixed feeding rather than exclusive breastfeeding or complementary feeding because they believed the chances of their child contracting HIV was low.

*“… mostly mothers did not care for their children. Seriously, even when their status is disclosed, they deny ART for their children and refuse them getting HIV test … they also practice mixed feeding …”* HP7

Apathy was also apparent among health care professionals lack of drive for the program and patient-centeredness. Health care professionals often collected incomplete history, inconsistency in prescribing medicine and conducting required investigations. There was a lack of strict guidelines on medication administration, follow-up care on children and when to test them despite the WHO guidelines being available. Also, there was no clear hospital/center record on when the mothers should come for follow-up; therefore, when a mother misses her appointment; it takes time to know about it. Also when it is apparent that the mother is lost to follow-up, healthcare professionals did not usually call the mothers despite having access to their contact details, phones to call and even transport to check on the patients in their villages when necessary.

*“… .she has not prescribed medications like co-trimoxazole if the child looks healthy most of the time I was not sending the sample to the laboratory if the mothers did not complain that their children had gastrointestinal, genitor-urinary, and other sign of illness …” HP12*

### Stigma and discrimination

*“… the emotion of my family is variable, and they discriminate and condemn me as a guilty. Thus, I am not interested and focused on the follow-up; rather I worried about the view and opinion of my family …” M10*

Stigma and discrimination were identified as a major hurdle for parents to continue with the PMTCT and also to adhere to exclusive breastfeeding or formula feeding leading to the HIV infection of the children. It was also indicated as a direct cause of mortality due to the lack of testing of children by parents despite knowing the risk the child faces. In Amhara region, stigma and discrimination emanate from the lack of acceptance by the family and lack of understanding by the health professionals.

*“… When I was going to the hospital for follow-up, the reactions of health professionals are not welcoming. Sometimes, health professionals closed the PMTCT clinic, and sometimes they show uncomfortable facial expression while I ask them to provide the necessary care …” .*M6

Therefore, mothers do everything in their powers not to disclose their status or allow their children to be tested for HIV. To ensure that their status is not disclosed, mothers also practised mixed feeding or breastfeeding beyond the recommended time. The stigma and discrimination are also exacerbated by the lack of privacy when breastfeeding and father’s uncooperativeness and negligence about their own HIV status. Also, the lack of understanding of mothers and children needs and challenges by the health professionals leads to stigma and discrimination tendencies despite ethical conducts guidelines being in place

*“… when I oppose breastfeeding for the child, all the families pointed towards me as are you HIV positive or. Thus, I forced to continuing breastfeeding till 2 years old of my child unless I was risky for discrimination …”* M14

Health providers also identified stigma and discrimination as a major risk for LFTU and HIV child mortality in the region. Health workers stated that due to fear of stigma and discrimination many mothers do not opt for the PMTCT program and those that do, some do not complete the program due to fear and as such exposing children through mixed feeding and eventual not showing up for testing while the virus is affecting the baby if he/she is HIV positive.

*“… if mothers disclosed their status, the communities, including families, discriminate them in different events like delivery of first aid measures if mothers faced accident that cause bleeding, and annual cultural celebrations…”*HP3

### Access

Access to services was identified as one major factor in LTFU and child mortality due to HIV. Access to service theme includes the physical structure of the health facilities, resource availability, and distance to ART services for patients. The physical structure of health facilities was identified as a major hurdle to the privacy of patients and as such lead to patients not willing to participate in the PMTCT program. For instance, the lack of counseling room in majority of PMTCT facilities was cited as a major reason for LTFU and parents’ unwillingness to bring their babies for testing.

*“… when I visit the clinic for ANC service, they transferred me to another room that is labeled as PMTCT room … thus, it might be good if all the services provided for women handled in a secret room without label if we want to keep women’s status a secret …”* M18

*“… The structure of the clinic might not respect the privacy and confidentiality of the mother who visits the clinic. Mostly the room is arranged in nearby to ANC rooms. Thus, if mothers are positive, they are referred to PMTCT room which might be considered as discrimination for mothers …”* HP16

Also, resource availability was identified as a major barrier towards access to the PMTCT program and often led to parents losing trust in the program and abandoning it. Both parents and health care professionals cited the chronic shortage of PMTCT service providers. The shortage of healthcare providers often leads to long waiting periods, long queues for services which often exacerbate suspicion from families and friends hence leading to stigma and discrimination. This shortage also affects the women’s contribution to their homestead and further putting them into poverty.

*“… when we visit the clinic, the professionals are not available in the clinic, and we forced to turn back home without receiving the care and booking the next appointment …”* M21

*“… currently the attention towards PMTCT service is not good as previous. Some managers think the unit as an extra duty not as a routine duty. They did not assign sufficient staff in the PMTCT room, even when the hospital has many professionals. Furthermore, the staffs assigned in the PMTCT room did not receive any training …”* HP22

Shortage and interrupted supplies of medicines were also identified as a major reason for LTFU and child mortality due to HIV. Shortage of medicines used in Option B+ program was identified as a major issue as some centers can go for some months without essential drugs such as Nevirapine leading to patients being referred to other centers. When patients are referred to other centers’, sometimes they get confused about where to continue with the services, and some do not go but rather go home leading to LFTU and eventually child infection and possible deaths. As one ART officer stated, the absence of medications like Nevirapine cause referring patients to other ART centers, but some patients were not going to other ART centers and consequently went back home

*“… Most of the time the health care professionals tell us that medicine is not available, and instructs us to visit other centers …”* M9

*“… medicines particularly syrup preparations are frequently out of stock in ART centers’, and patients get tired on checking or being referred to other centers’, and they usually do not come for follow up...”*HP19

Lastly, participants, particularly mothers, indicate that distances to ART centers are a huge problem for many of them. Also, ART centers were reported to be isolated from other services so patients cannot get comprehensive health services and had to go to other services for those services.

*“… Some of the women are coming out of the town, which is far more than 10 km. This is a challenge in particular when medications are not there, and appointments moved to other days …”* M11

*“… According to the minister of health current PMTCT structure, the service is provided for the community in the cluster that exposes some mothers for long journey …”* HP15

### Healthcare providers’ attitudes

*“…professionals assigned in the PMTCT department are not involved in other services, and therefore not benefiting from some of the incentives others have. Also, the professionals are part of the community in which cultural practices are ingrained, and HIV and sexuality topics are a taboo; therefore, they might display negative attitude to these women based on myths or cultural perspectives. Consequently, the quality of health service provided for these women is usually compromised…”*HP9

Participants also indicated that healthcare providers often display negative attitudes toward PMTCT service consumers and also lack the commitment to the program. The negative attitudes and lack of commitment to the program have led some users to develop distrust in the program hence dropping or stopping to use the services altogether. Health care providers’ lack of interest in the program and welfare of the participants also lead some users who have lost-to follow-up does not seek services when the child gets ill or getting the child tested leading to complications and sometimes death.

*“…The facial expression and gestures of health professionals while you visit OPD or other health care service and PMTCT service are different. The PMTCT staffs deny our interests; they do not want to listen about the side effects of the drugs, and cannot accept other laboratory investigations requests…”* M2

*“…The PMTCT service provision culture needs to be modified. The mothers’ expectations when they visit PMTCT service, are ideal. In opposite, the hospitals did not fulfill the requirement for the services as they advertise them to the mothers. Thus, the burden is for the PMTCT professionals, and would have a negative attitude as a reaction for the mothers’ expectations and hospitals preparation…”* HP1

### Social determinants of health

*“…the social determinants like poverty, lack of husband support, and other familial responsibilities placed a burden on women, which are repeatedly reported from mothers as a cause to LTFU in the program and latter cause to child mortality.”* HP19

Social determinants theme emanated from reports of poverty and socio-cultural challenges facing women in the PMTCT program such as poverty and lack of involvement of spouses. Poverty was identified as a major factor in both women loss-to follow-up and child mortality related to HIV. Poor women often did not have access to services due to distance, competing priorities of feeding their families and attending PMTCT programs. Poverty was also highlighted as a major contributor to poor nutrition and lack of care that exposes the child to many illnesses, including death.

*“…Mothers from a poor household might face multiple problems. The woman might become malnourished and fasten the progress of the [HIV] infection, which might also increase the risk of the transmission to the child, or the woman might busy on finding jobs to feed their family. The child might become undernourished and develop other infections. All such scenarios might cause mothers to lose hope on life and might discontinue the PMTCT service…”* HP4

Also, socio-cultural issues relating to lack of spousal involvement in maternal and child health issues were evident as contributors to LTFU and child mortality and mothers decried the lack of spousal involvement as one reason. Most women and health professionals stated the long-held cultural practices in the country aid husbands to neglect children and mothers. Usually, husbands spent the working days from Monday to Friday away from home at work, and appointments of mothers for PMTCT are also during these working hours. Therefore, if there are responsibilities that require the presence of a family member in the home during the working days, the woman is forced to discontinue the follow-up, unless there is a provision where the husband can miss work to supports them.

*“…The cause for the infection is my husband, but he always claims it is me. He has not agreed on the follow-up and is not willing to visit the clinic together with me. He forced me to discontinue the care; I refused and took precautions for my child…”*M3

*“…I am not sure about the source of infection, but my husband always blamed me, and he thought as I am the source of infection. Thus, he is thinking that neglecting me means just punishing me for my faults. He frequently insults me and also uses physical force on the days of my follow up to prevent from visiting the clinic…”*M20

### Recommendations for reduction of loss-to follow-up and child mortality

Recommendations for reduction follow-up and child mortality is divided into four themes; 1) Access - addressing improvement of physical barriers, resource availability and mechanisms for follow-up, 2) Psychosocial support- capturing avenues to avail psychosocial support and building community networks for resilience, 3) Education and awareness- addressing parental and healthcare providers awareness on issues of loss-to follow-up, child mortality and making PMTCT facilities clients focused, 4) Empowerment- elucidating on areas that could be tapped into to reduce disparities and address social determinants of health.

## Access

Access theme under improvement of PMTCT aimed at eliminating both physical barriers, resource shortages, improving quality of services and devising mechanisms for follow-up. While participants acknowledged the limitation of the physical barriers, they understood that building more structures comes at a cost; therefore, they advocated re-designation of spaces to improve patient flow.

*“…the ART pharmacy, and follow up OPD is a separate building, which is a challenge for a woman who has not disclosed their HIV status. Thus, if the pharmacy and follow up of HIV positive mothers are hidden, it respects their privacy and confidentiality, particularly who are not disclosed their status…”*M7

Participants also indicated that ethical issues need to be addressed in counseling and health care providers be trained in sensitivity of HIV issues and how they can best support the mothers during these difficult times. HIV officers also stated the unethical conduct of professionals was one of the reasons for mothers’ loss to follow up and other consequence of LTFU.

*“…One health professional insulted me using a strong term that related to HIV. Most health professionals are also thought that all the woman who acquire HIV because of their sexual misconducts and consider them as whores…”*M23

For shortage of drugs and interruption of supplies, participants suggested that health care providers form a network so that they know where clients can get the services or even have the drugs shipped to areas where they are rather than referring the patients to other centers.

*“…When mothers come to take medicine, and we fail to give them, we feel sorrowful, because we understand the challenges they face, particularly if they have not disclosed their status. Thus, I recommend the centre should borrow medicine from nearby centers or call the centers to notify them on the mothers expected to visit and facilitate an appointment for them or make an appointment for other days if the medicine is not available around the communities…”*HP21

Participants also advocated for the centers to be proactive and develop a mechanism to trace patients to improve children’s outcomes rather than just labeling them lost to follow-up. Some of the suggested mechanisms include phone-based communication, group-based communication or reaching out to the patient contact person.

*“…Most of the health professionals who are working in the PMTCT department are not checked who lost the follow up regularly. If the PMTCT staffs call before a couple of days of appointment to show respect and degree of care, the mothers might visit timely. Also, the professionals or health facilities should consider different tracking mechanism starting phone communication to group communication and then contact person, who may be closed person…”* HP7

### Psychosocial support

Psychosocial support was identified as a solution to fight against stigma and discrimination and carelessness of the mothers. Avenues identified include building social networks for support and resilience as well as involving other family members in sexual and reproductive health of the mothers. Social networks identified include support groups such as mother –to- mother association and volunteers visiting mothers in homes to provide both emotional support and assistance to mothers.

*“…The mother-to-mother association is significantly important in breaking the negative community perceptions about HIV positive people in different methods. For example, when mothers visit HIV positive mothers frequently, care for the children born to HIV positive mothers, and teach the people who hold negative thought about HIV positive mothers…”*M24

*“…The social networks and civic associations like mother-to-mother association, and other charity associations have a significant role in returning HIV positive mothers to the community, improving their economy, family and social life… .”*HP16

Improving the quality of counseling to broader aspects of the woman’s life was also identified as possible interventions for loss to follow-up and prevention of child mortality. Also training health workers to be sensitive to the needs of the women was identified as a form of support that could encourage more women to join the program and keep the ones that have joined the program until the end.

*“…the quality of counseling, including eye contact, facial expression, and other body gestures are very important to retain mothers to the end of the program. Such quality among PMCT professionals would have occurred through frequent training …”* HP17

*“…Most PMTCT professionals who work in health facilities are not trained; the training was given for former staff; Thus, we are working with our basic education; therefore, training should be given periodically …”*HP20

### Education and awareness

The health and awareness theme focuses on improving the healthcare-seeking behavior of parents to improve treatment adherence and also continued screening of children in the postnatal period to reduce deaths and LTFU. Education and awareness will also open avenues for other family members, particularly spouses, to be involved in maternal and child health.

*“… if mothers disclosed their status to their husbands or other family members, it would be good to ensure that education is also provided for the family and husbands because they will remind the mothers about follow-up care when they do not go …”* HP2

### Empowerment

Empowerment theme came as a response to social determinants such as poverty as a major contributor to LFTU and death. Participants suggested that women should be empowered through civic societies to mitigate the effects of poverty. Most health professionals who work in hospitals and HIV officers stated that Mothers association and other civic societies are essential to prevent mothers and child LTFU.

*“… mothers association is very important in which mothers can visit HIV-positive mothers in their homes, arrange financial support and motivate them to complete their follow up and may also provide care for their children during their visit, which encourages mothers to accomplish the follow-up and prevent death…”*HP3

## Discussions

This study adds to the existing evidence of factors that contributes to LTFU but bring a new perspective by looking at both providers and mothers as well as addressing possible reasons for child mortality in addition to LTFU among this group. The study also gives a unique view of factors that contribute to LTFU and child mortality due to HIV at a district level, using multi-sites and suggests community informed multifaceted interventions that can be adopted to improve the retention of mothers and children in option B+ and ultimately reduce mortality of children due to HIV.

The results of this study demonstrate that multi-factors play a role in mothers choice of continuation with PMTCT services and are similar to studies in Gomba district, Uganda [22], in Cote-d’Ivoire [37], in Malawi [38] and Malawi and Uganda [39]. The themes of stigma and discrimination, social determinants and partial access reported in this study are shared across these studies as major barriers to retention in PMTCT program [22, 38–41].

Stigma and discrimination are reported as a major barrier across studies and countries, and it was reported as a major issue by the participants including internalized stigma [22, 38, 39, 41, 42]. Although the information on HIV is widely available through all media; this study confirmed that stigma and discrimination are still a significant issue in HIV in Ethiopia and other African states stigma [22, 38, 39, 41, 42]. Unlike findings from Schechter et al. [41] stigma in Amhara region is not largely internalized but rather from the community and sometimes health care professionals.

The social determinants of poverty, cultural taboos, and lack of spousal support as important barriers for women to access services or continue with services was also a critical result in this study. It is also compounded the struggle with stigma and discrimination as some women would choose to protect their relationships through leverage of husbands’ absence at Antenatal care and cultural practices that would raise a flag in mothers do not breastfeed the child. Similar results were reported elsewhere [39] as their main finding in Malawi and Uganda populations and Malawi [42]. Poverty as a barrier reported here has also been reported by other studies as a lack of funds to travel for Antenatal care [22, 41].

Access is reported as a major barrier in this study focusing on structure, disruption of the supply of medications, and distance of ART centers. Other studies reported on this theme, focusing mainly on the distance of ART centers and the cost of care [22, 41, 43]. This study brings in a new perspective to the access theme by highlighting how the physical structure and labeling of the buildings perpetuate the self-stigma and discrimination which lead to LTFU except in a study by Bwirire et al. [41] where they alluded to the long quest at ANC as a reason for LTFU. Also, this study brings in a system issue on the supply of ART drugs in the region which has not previously reported by other studies addressing LFTU in sub-Saharan Africa [28, 44, 45].

The study findings also reported on the theme of apathy and healthcare professionals’ attitudes. The findings of carelessness are opposite those reported by Kiwanuka et al. [22] and Schechter et al. [41] where participants were reported to see participation in PMTCT program as a positive attribute for hope, being alive and be able to raise an HIV free or healthy child. In this study, mothers were reported not to care for their children’s outcomes and often did not bring children to health centers when sick despite knowing that they may be HIV exposed. Such behavior was also reported by Kebede and Taye [45] in the Amhara region, where they reported that mothers who attended ANC reduced chances of their child infection compared to those who did not. The healthcare professionals’ attitudes are mostly not alluded to in literature within the region but were reported to be critical to ensure the success of the PMTCT program in Brazil [46].

The interventions suggested by the participants have also alluded as successful measures in other sub-Saharan Africa countries. For instance, Geldsetzer et al. [47] in their review emphasis that most of LFTU interventions are successful in sub- Saharan Africa including phone calls and text messages to remind clients to come back for services. The peer, community, layperson support system has been implemented in Uganda and also showed an increase in retention among HIV positive mothers on follow-up care [48]. However, results from [37] in Zimbabwe shows that peer support group did not show any difference in retention of mothers.

### Limitations

The study has some limitations. The first limitation is the failure to include participants who have been already LTFU during the study, which could have given the first-hand experience why they stopped coming for the services. Getting this individual to participate in the study was difficult as they were not willing to be associated with the Option B+ program. Also, some participants may have responded to interviews based on social desirability, which is inherent to such designs. Since the data were collected in Amharic; some of the contexts might not be represented well in English or lost in translation while the translation was verified.

## Conclusions

Loss-to-follow-up is a critical barrier to successfully raising HIV free generation and reducing AIDS-related child mortality in Amhara Region, Ethiopia. Both mothers and healthcare providers identified apathy, lack of access, stigma and discrimination, healthcare professionals’ attitudes and social determinants as major determinants to LTFU and child mortality. While psychosocial support through the peer support group, women empowerment, improvement of access to services including a constant supply of medications, physical planning of buildings, engagement of civic societies and education and awareness are considered possible interventions to reduce LTFU and child mortality. Therefore, a multi-stakeholder approach, including structural changes are required to support women and their children to ensure that individuals, communities and country enjoy the full benefits of option B+ and lead to an HIV free generation.

## Supplementary Information

**Additional file 1.**

## Data Availability

The raw materials that support the conclusion of this research will be available to researchers needing the data to use for non-commercial purposes through requesting the authors through their e-mail.

## Abbreviations

LTFU: Loss to follow up
HIV: Human immune virus
ART: Anti-retro viral therapy;
PMTCT: Prevention of mother to child transmission
ANC: Anti-natal care
OPD: Outpatient department
M: Mother
HP: Health professional
AIDS: Acquired immune deficiency syndrome
SDG: Sustainable development goal
WHO: World Health Organization.

## References

[1] Ki-moon B (2015). The millennium development goals report.

[2] Joint U (2016). Programme on HIV/AIDS. Prevention gap report.

[3] HIV/AIDS JUNPo (2019). UNAIDS 90–90-90 an ambitious treatment target to help end the AIDS epidemic.

[4] Buse K, Hawkes S (2015). Health in the sustainable development goals: ready for a paradigm shift?. Glob Health.

[5] WHO (2018). Treatment of children living with HIV.

[6] UNICEF (2020). Global and Regional trends.

[7] UNAIDS. Data 2019. Available from http://rstesa.unaids.org/documents/publications/77-2019-unaids-data-en/file.

[8] UNAIDS (2019). J., country’s epidemiological fact sheet. ETHIOPIA | 2016.

[9] UNAIDS, Ending AIDS: progress towards the 90-90-90 targets (2017). Global AIDS update.

[10] Sibanda EL, Weller IVD, Hakim JG, Cowan FM (2013). The magnitude of loss to follow-up of HIV-exposed infants along the prevention of mother-to-child HIV transmission continuum of care: a systematic review & meta-analysis. Aids.

[11] UNAIDS (2020). Ethiopia.

[12] WHO (2012). Use of antiretroviral drugs for treating pregnant women and preventing HIV infection in infants.

[13] World Health Organization. Towards universal access: Scaling up priority HIV/AIDS interventions in the health sector: progress report 2010: WHO; 2020. Available from https://www.who.int/hiv/pub/2010progressreport/en/.

[14] UNAIDS (2020). On the fast-track to an AIDS free generation. The incredible journey of the global plan towards the elimination of new HIV infections among children by 2015 and keeping their mothers alive.

[15] Kibret GD, Ferede A, Leshargie CT (2019). Trends and spatial distributions of HIV prevalence in Ethiopia. Infect Dis Poverty.

[16] Endalamaw A, Demsie A, Eshetie S (2018). A systematic review and meta-analysis of vertical transmission route of HIV in Ethiopia. BMC Infect Dis.

[17] Umede-Moyo S, Filteau S, Munthali T, Todd J, Musonda P. Implementation effectiveness of revised (post-2010) World Health Organization guidelines on prevention of mother-to-child transmission of HIV using routinely collected data in sub-Saharan Africa: a systematic literature review. Medicine. 2017;96(40). 10.1097/MD.0000000000008055 PMC5737996 28984760

[18] Tolossa T, Kassa GM, Chanie H (2020). Incidence and predictors of lost to follow-up among women under option B+ PMTCT program in western Ethiopia: a retrospective follow-up study. BMC Res Notes.

[19] UNICEF (2018). Addressing the global HIV epidemic among pregnant women, mothers, children and adolescents UNICEF’s global HIV response 2018–2021.

[20] Kyaw K, Oo M, Kyaw N (2017). Low mother-to-child HIV transmission rate but high loss-to-follow up among mothers and babies in Mandalay, Myanmar; a cohort study. PLoS One.

[21] Bigirimana F, Owiredu MN, Nuwagira IB (2016). Prevention of mother-to-child transmission technical update: implementing the ‘treat all’ approach among pregnant and breastfeeding women living with HIV in the WHO African region.

[22] Kiwanuka G, Kiwanuka N, Muneza F (2018). Retention of HIV infected pregnant and breastfeeding women on option B+ in Gomba District, Uganda: a retrospective cohort study. BMC Infect Dis.

[23] Lumbantoruan C (2018). Understanding women’s uptake and adherence in option B+ for prevention of mother-to-child HIV transmission in Papua, Indonesia: a qualitative study. PLoS One.

[24] Lincoln Y, Guba E (2010). Naturalistic Inquiry. 1985 Newbury Park.

[25] Polit DF, Beck CT. Nursing research: generating and assessing evidence for nursing practice: Lippincott Williams & Wilkins; 2008.

[26] Negash TG, Ehlers VJ (2016). An assessment of the outcomes of prevention of mother-to-child transmission of HIV services in Addis Ababa, Ethiopia. Curationis.

[27] Elo S, Kyngäs H (2008). The qualitative content analysis process. J Adv Nurs.

[28] Burnard P (1991). A method of analysing interview transcripts in qualitative research. Nurse Educ Today.

[29] Downe-Wamboldt B (1992). Content analysis: method, applications, and issues. Health Care Women Int.

[30] Schreier M. Qualitative content analysis in practice: Sage publications; 2012.

[31] NVivo Q. QSR International Pty Ltd. Doncaster, Victoria, Australia. 2002.

[32] Saldaña J. The coding manual for qualitative researchers: Sage; 2015.

[33] Patton MQ (2002). Qualitative research and evaluation methods.

[34] Conrad P (1987). The experience of illness: recent and new directions. Research in the sociology of health care Vol 6: the experience and management of chronic illness.

[35] Morse JM, Barrett M, Mayan M, Olson K, Spiers J (2002). Verification strategies for establishing reliability and validity in qualitative research. Int J Qual Methods.

[36] Lincoln Y, Guba E. Naturalistic inquiry 1985 Newbury Park. California: Sage Publications [Google Scholar]; 2010.

[37] Foster G, Orne-Gliemann J, Font H, Kangwende A, Magezi V, Sengai T (2017). Impact of facility-based mother support groups on retention in care and PMTCT outcomes in rural Zimbabwe: the EPAZ cluster-randomized controlled trial. J Acquir Immune Defic Syndr.

[38] Bwirire L, Fitzgerald M, Zachariah R, Chikafa V, Massaquoi M, Moens M (2008). Reasons for loss to follow-up among mothers registered in a prevention-of-mother-to-child transmission program in rural Malawi. Trans R Soc Trop Med Hyg.

[39] Flax VL, Yourkavitch J, Okello ES, Kadzandira J, Katahoire AR, Munthali AC. “If my husband leaves me, I will go home and suffer, so better cling to him and hide this thing”: The influence of gender on Option B+ prevention of mother-to-child transmission participation in Malawi and Uganda. PLoS One. 2017;12(6):e0178298. 10.1371/journal.pone.0178298 PMC5464556 28594842

[40] Cataldo F, Seeley J, Nkhata MJ, Mupambireyi Z, Tumwesige E, Gibb DM (2018). She knows that she will not come back: tracing patients and new thresholds of collective surveillance in PMTCT option B+. BMC Health Serv Res.

[41] Schechter J, Bakor AB, Kone A, Robinson J, Lue K, Senturia K (2014). Exploring loss to follow-up among women living with HIV in prevention of mother to child transmission programmes in cote d’Ivoire. Glob Public Health.

[42] Cataldo F, Chiwaula L, Nkhata M, van Lettow M, Kasende F, Rosenberg NE (2017). Exploring the experiences of women and health care workers in the context of PMTCT Option B Plus in Malawi. J Acquir Immune Defic Syndr (1999).

[43] Tweya H, Gugsa S, Hosseinipour M, Speight C (2014). Ng’ ambi W, Bokosi M, et al. understanding factors, outcomes and reasons for loss to follow-up among women in option B+ PMTCT programme in Lilongwe, Malawi. Tropical Med Int Health.

[44] Kassaw MW, Abebe AM, Abate BB, Getu MA, Zemariam AB. Mother to child HIV transmission and association among exposed infants after option B+ guideline implementation in the Amhara regional state referral hospitals, Ethiopia. Ethiopia. Int J Infect Dis. 2020. 10.1016/j.ijid.2020.03.006 32247052

[45] Tigabu Z, Wasie B. Outcomes and linkage to chronic care of HIV exposed infants among health centers and hospitals in Amhara region, Ethiopia: implications to prevention of mother-to-child transmission of HIV program: a cross-sectional study. Pan Afr Med J. 2016;24(1). 10.11604/pamj.2016.24.61.7305 PMC5012797 27642402

[46] da Cruz Gouveia PA, da Silva GAP (2014). Predictors of loss to follow-up among children registered in an HIV prevention mother-to-child transmission cohort study in Pernambuco. Brazil. BMC Public Health..

[47] Geldsetzer P, Yapa HMN, Vaikath M, Ogbuoji O, Fox MP, Essajee SM (2016). A systematic review of interventions to improve postpartum retention of women in PMTCT and ART care. J Int AIDS Soc.

[48] Namukwaya Z, Barlow-Mosha L, Mudiope P, Kekitiinwa A, Matovu JN, Musingye E (2015). Use of peers, community lay persons and Village Health Team (VHT) members improves six-week postnatal clinic (PNC) follow-up and Early Infant HIV Diagnosis (EID) in urban and rural health units in Uganda: A one-year implementation study. BMC Health Serv Res.

