# Supplementary material for: The perceived determinants and recommendations by mothers and healthcare professionals on the loss-to-follow-up in Option B+ program and child mortality in the Amhara region, Ethiopia
Source: BMC Infect Dis. 2020 Nov 23;20:876. doi: 10.1186/s12879-020-05583-6 (PMC7686694; doi:10.1186/s12879-020-05583-6)
Supplement: Supplementary file 1 — Additional file 1. [file 12879_2020_5583_MOESM1_ESM.docx]

**Interview Guide-English version**

Thank you for participating in our study that looks at your opinion and experiences regarding mothers who drop from the PMTCT program in our region. We are not checking on anybody; in fact, our discussion is strictly for research purposes and is not reported to your healthcare providersor employers. Therefore, what you say will not affect your care/job or relationship with people you work with. If you feel uncomfortable with our discussion,you can opt not to answer some of the questions, andyou can always ask us to stop anytime. There will be no penalties againstyou. This interview may last about 2.5 hours. Now let us begin.

**Yourself:**Tell me a little about yourself and your family

____________________________________________________________________________________________________________________________________________________________

Probe mothers: Where do you live? How many are you in the household?

Probe health care professionals: How long have you worked here? Where are you from? Do you have any children of your own?

**Pregnancy/Job:** How is your pregnancy so far/how is your work?

____________________________________________________________________________________________________________________________________________________________

Probe mothers: How would you describe your pregnancy? Is it something that you expected? If so, what was the expectation? If not, tell me what went wrong.

Probe Health care professionals: How would you describe your everyday job? Is it something that you expected? If so, what were those expectation? If not, tell me where it falls short. Would you consider yourself to be enjoying your work?

____________________________________________________________________________________________________________________________________________________________

**Mother understanding of PMTCT:** Tell me about what you know about the PMTCT program that you are participating in

____________________________________________________________________________________________________________________________________________________________Probe: Is there anything that you have noticed about the program that is different from your other experiences in other pregnancy or in other health services. What comes to your mind when you think about the program [used common Amharic word for program]. How well do you think the program protect you and your child?

**Healthcare providers understanding of PMTCT program**: Tell me about the PMTCT program here at your hospital or zonal office.

____________________________________________________________________________________________________________________________________________________________

Probes: What have you recognized about it? Has it changed over time? What do you think about the services that you offer? What has been going well and frustrating about it? How can it be improved?

**Contributors to LFTU Mothers**: Have you experienced or witnessed a woman who stopped using the PMTCT services?

____________________________________________________________________________________________________________________________________________________________

Probes: How often do you think this happens? What do you think caused you or these women to stop using the PMTCT services? What other reasons that exist in the community, hospital that can make you stop you from continuing your PMTCT services? Do you see yourself one day stopping using these services? [Probe gentle as it may cause discomfort for individuals who have experienced LFTU

**Contributors of LFTU Healthcare providers:** Have you experienced or witnessed women stopping or being lost to follow-up in the PMTCT program?

____________________________________________________________________________________________________________________________________________________________

Probe: How common is this problem in your practice? What reasons have these women advanced about dropping off the program? What do you think contributes to these women stopping to using the PMTCT services? What other reasons do you think to stop these women from continuing with the program in the community, hospital, etc.?

**Consequences of LFTU Mothers**: What do you think happens to the mothers that stop using the program

____________________________________________________________________________________________________________________________________________________________

Probe: What do you think happens to the health of these women? What happens to the health of their children? How do you think these women are viewed in the community?

**Consequences of LFTU healthcare professionals:** What usually happens to the women who are lost to follow-up?

____________________________________________________________________________________________________________________________________________________________

Probe: Do they ever come back to the hospital for the same or different services? How are their health outcomes? What is the often health outcomes of their children? How much do you think to contribute to children deaths?

**Prevention of LFTU mothers:** What do you think could be done to prevent women from stopping the use of the program once there have started?

____________________________________________________________________________________________________________________________________________________________

Probe: What do you think the mothers should do? The families? The community? The health care providers, The physical structures, What parts of the program that should be reviewed? Moreover, what do you suggest should be done?

**Prevention of LFTU healthcare providers**: What do you think should be done to prevent women from being lost -to follow-up?

____________________________________________________________________________________________________________________________________________________________

Probe: What do you think the mothers should do? The families? The community? The health care providers, The physical structures, What parts of the program that should be reviewed? And what do you suggest should be done?

Where do you see the PMTCT program in the future?

______________________________________________________________________________________________________________________________________________________________________________________________________________________________________________________________________________________________________________________________________________________________________________________________________

Thank you for your time and helping us understand why women stop using PMTCT services and how it affects the mother and child health. We believe your answers will go a long way in helping us understand the PMTCT program and how it falls short and how we can best work towards improving it. Our interviews will end here. Once again thank you.
